# Caesarean section and adiposity at 6, 18 and 30 years of age: results from three Pelotas (Brazil) birth cohorts

**DOI:** 10.1186/s12889-017-4165-3

**Published:** 2017-03-14

**Authors:** Aluisio J. D. Barros, Leonardo Pozza Santos, Fernando Wehrmeister, Janaina Vieira dos Santos Motta, Alicia Matijasevich, Ina S. Santos, Ana M. B. Menezes, Helen Gonçalves, Maria Cecília Formoso Assunção, Bernardo L. Horta, Fernando C. Barros

**Affiliations:** 1 Post-graduate Program in Epidemiology, Federal University of Pelotas, Rua Marechal Deodoro, 1160 – 3o piso, 96020-220 Pelotas, Brazil; 2 Federal University of Pampa, Itaqui, Brazil; 3 Post-graduate Program in Health and Behaviour, Catholic University of Pelotas, Itaqui, Brazil; 4 Department of Preventive Medicine, School of Medicine, University of São Paulo, São Paulo, Brazil

**Keywords:** Cesarean section, Body composition, Adiposity, Cohort studies

## Abstract

**Background:**

Association between caesarian section (C-section) and obesity is controversial and mostly based on body mass index (BMI), which has inherent limitations. Using direct estimates of body fat mass, we aimed to assess the association between C-section and adiposity using fat mass index and BMI z-score in three birth cohort studies from Pelotas, Brazil.

**Methods:**

We measured weight, height and fat mass (using dual X-ray absorptiometry (DXA)) at ages 6, 18 and 30 years among participants in the 2004, 1993 and 1982 population-based Pelotas Birth Cohort Studies, respectively. We used multiple linear regression analysis to examine the crude and adjusted association between C-section and the body composition indicators. We also modelled height as an outcome to explore the presence of residual confounding.

**Results:**

We observed that fat mass index and BMI z-score were strongly and positively associated with C-section in the crude analysis. However, when we adjusted for socioeconomic characteristics, maternal BMI, parity, age and smoking during pregnancy, effect estimates were attenuated towards the null, except for 30-year-old women. In those women from the 1982 cohort, C-section remained associated with fat mass index (β = 0.82; CI95% 0.32;1.32) and BMI z-score (β = 0.15; CI95% 0.03;0.28), even after adjusting for all potential confounders, suggesting an increase in fat mass index and BMI at 30 years among those born by C-section.

**Conclusion:**

We found no consistent association of C-section with fat mass index measured by DXA and BMI z-score in individuals aged 6, 18 and 30 years, except for women in the latter group, which might be explained by residual confounding. Confounding by socioeconomic and maternal characteristics accounted for all the other associations.

## Background

Obesity and metabolic disorders are increasing around the world [1, 2]. In Brazil, in the last three decades, the prevalence of obesity in adults increased from 2.8 to 12.4% in men and from 8 to 16.9% in women [3]. Worldwide, obesity is one of the main targets for public health interventions. As such, any potentially modifiable risk factor related to obesity is of high priority for the public health agenda [4].

In recent years, several studies reported an association between C-section and increased BMI or higher risk of obesity [5–11]. The biological proposed mechanism linking C-section and obesity is related to changes in the gut microbiota that may be caused by use of antibiotics or by not going through the birth channel [6, 12].

C-section rates are increasing globally, accounting for more than one third of all births in some countries such as USA and China [13]. In Brazil, a recent national survey showed that C-section deliveries are more common than normal births (54.7% of C-section births in 2013) [14]. In many countries, C-section births are strongly associated with wealth and coverage by private health insurance [15, 16]. Because obesity is commonly associated with socioeconomic position (SEP) indicators, confounding is a reasonable alternative explanation for the reported associations between C-section and obesity. This might be the reason why some studies exploring this association failed to demonstrate it after adjustment for confounders [12, 17, 18].

BMI is a measure of body size and has been used in practice to define overweight and obesity. However, despite presenting high correlations with adiposity, BMI is not a direct measure of body fat. Given that we recently evaluated the body composition from participants of our three birth cohorts, including direct estimates of body fat mass, we can advance the study of the potential association between C-section and obesity by analyzing the direct association between body fat (as measured by the fat mass index) and C-section. Therefore, we aimed to study the association between C-section and adiposity using fat mass index and BMI in individuals aged 6, 18 and 30 years adjusting for a range of potential confounders.

## Methods

### Subjects

Pelotas is a city located in Southern Brazil, with a population of approximately 330,000 inhabitants according to the 2010 national census. Three birth cohorts are currently being followed-up in the city, starting in 1982, 1993 and 2004. They share a very similar methodology – in their reference year, all newborns whose mothers lived in the urban area of the city were recruited to the studies. Pelotas had four maternity hospitals in 1982 and five in 1993 and 2004 that were monitored for births during the whole reference year of each cohort. Limiting recruitment to hospitals is not a problem in Pelotas since more than 98% of births are institutional, and those few mothers giving birth elsewhere usually go to a hospital for postnatal checks and care.

Specially trained field workers approached mothers in the first 24 h after giving birth and invited them to participate in the study. After consenting, mothers completed a questionnaire containing information about the family, the current pregnancy and delivery, and their babies were examined and had their length measured. Furthermore, field workers collected information about birth conditions, including Apgar score and weight, from hospital records. A full account of the methods used in the perinatal studies of these three cohorts was published previously [19].

The three birth cohorts recruited decreasing numbers of babies given the rapid decline in fertility observed in Brazil. In 1982, 5914 babies were included in the study, 5249 in 1993 and 4231 in 2004. In all cases, refusals to participate were below 1%. We followed up these three cohorts several times since recruitment, each with somewhat different assessment ages. Details for each cohort are available in specific methods papers [20–22].

### C-section and perinatal information

Directly relevant to this study, interviewers obtained the date and time of delivery, as well as type of delivery during the perinatal interview. We also obtained other variables used as potential confounders at this moment: quintiles of SEP, according to Brazilian National Economic Index (IEN) [23], an asset index based on household goods and the household head’s education; maternal age at birth (years); maternal education (years); maternal reported skin colour (white, brown or black, according to the classification used by the Brazilian Bureau of Census, IBGE); parity (1, 2, 3 or ≥4 siblings); smoking during pregnancy (0, <20 or ≥20 cigarettes per day); pre-gestational BMI (based on reported weight before the pregnancy and measured height at the interview); financing of delivery (private insurance vs free public health system); and the child’s birth weight and length (except for 1982 birth cohort, where birth length was not measured).

For the 2004 cohort, we used maternal BMI obtained at the 3-month follow-up (based on height and weight measured during the assessment) instead of pre-gestational BMI since height was missing for about one third of the sample. The correlation between pre-gestational and 3-month follow-up BMI was 0.86 and Lin’s concordance coefficient [24] was 0.82, showing good agreement for those women with complete data.

### Anthropometric and body composition information

The latest follow-up of each cohort included a detailed assessment of body composition. We measured participants’ body fat, lean and bone-mineral masses at the research clinic using dual-energy X-ray absorptiometry (DXA; GE Lunar Prodigy densitometer) in a full-body scan. Specially trained technicians carried out the exams, with participants in supine position using light and tight-fitting shorts and sleeveless tops. We asked participants to remove all metal accessories, such as bracelets, earrings or piercings. The examiners assessed the quality of DXA exams with participants still on the machine and repeated it, if necessary.

Height was measured twice by trained technicians using a Harpenden metal stadiometer, with 1 mm precision (Holtain, Crymych, UK). We used arithmetic mean of two measurements. We assessed weight using a high precision scale (0.01 kg), part of the BODPOD machine (Cosmed, Italy, http://goo.gl/7jzfLc) used for further body composition assessments. For each participant, we collected all measures included in this analysis on the same day.

Due to observed discrepancies between measured weight from BODPOD scale and total body mass from DXA, we adjusted some body composition indicators from DXA. Total body mass was obtained by adding up total fat, lean and bone-mineral masses. We calculated the percentage of fat mass by dividing fat mass by total body mass. We obtained adjusted fat mass by applying the DXA percentage of fat mass to the weight, as measured by the BODPOD’s scale.

We calculated fat mass index by dividing the adjusted fat mass (kg) by height (m) squared. For the 1993 and 2004 cohorts, we calculated the z-scores of BMI-for-age using the WHO 2007 growth reference [25]. For the 1982 cohort, we calculated BMI by dividing weight from BODPOD scale (kg) by the square of height (m), and afterwards we standardized the result to provide a similar scale used in the 1993 and 2004 cohorts.

### Statistical analyses

We used multiple linear regression analysis to examine the crude and adjusted associations between the three body composition outcomes and type of delivery. We adjusted for potential confounders in three steps: 1. we included SEP at birth, maternal schooling, mother’s skin colour and financing of delivery (public health system or private health system); 2. we included variables of level 1 plus pre-gestational maternal BMI, parity, maternal age at birth and smoking during pregnancy; 3. we included variables in level 1 and 2 plus birth weight and length.

Because SEP information is difficult to measure accurately and SEP is strongly associated with both C-section and adiposity in Brazil, residual confounding is an important concern. We thus did a parallel analysis using height as outcome to check for residual confounding. Since we do not expect any relationship between C-section and height, an association after adjustment would suggest residual confounding. For the three cohort studies, we used current height (cm), and the same multiple linear regression analysis as described above.

## Results

We included 3607 members from the 1982 cohort, 3961 from the 1993 cohort and 3317 from the 2004 cohort study, representing, respectively, those individuals followed-up at 30, 18 and 6 years who had available body composition information. In the three cohorts, the average age of mothers at birth was around 26 years (25.8 for the 1982 cohort, 26.1 for the 1993 and 26.2 for the 2004 cohort study), and 40% of them had just one child.

In the 1982 cohort, participants followed up at 30 years were more likely to be female, to be born preterm, and to have an intermediate SEP, when compared to those lost to follow-up. In the 1993 cohort, follow-up rates were higher among individuals with intermediate SEP, in individuals born preterm, and in those participants with low birth weight. In the 2004 cohort study, children lost to follow-up at 6 years had lower SEP and were exclusively breastfed for less time when compared to those followed at 6 years. There was no difference in birth weight and gestational age in the 2004 cohort.

The majority of mothers was white (Table 1), and maternal education improved over the 22 years covered by the three cohorts. Maternal overweight and obesity as well as coverage by private health system at birth increased, while birth weight remained stable. C-section rates increased steadily, from 27% in 1982 to 31% in 1993 and 45% in 2004. Finally, when we evaluated body composition indicators based on recent assessments, females presented higher fat mass index than males in the three ages studied (*p* < 0.001 for all cohorts) (Table 1).

**Table 1:** Characteristics of cohort members and their mothers at the time of birth and in the last follow-up of each cohort, by sex. The 1982, 1993 and 2004 Pelotas (Brazil) birth cohorts, 2010–2013

	2004 cohort		1993 cohort		1982 cohort	
	6 years		18 years		30 years	
	Male	Female	Male	Female	Male	Female
Past categorical variables (at birth)	N (%)	N (%)	N (%)	N (%)	N (%)	N (%)
Maternal skin colour						
White	1267 (74.2)	1166 (72.5)	1519 (77.3)	1529 (76.7)	1429 (81.5)	1538 (83.0)
Brown/Black	441 (25.8)	443 (27.5)	446 (22.7)	465 (23.3)	324 (18.5)	315 (17.0)
Maternal schooling (years)						
0–4	254 (15.0)	241 (15.2)	517 (26.3)	536 (26.9)	558 (31.9)	595 (32.1)
5–8	697 (41.1)	680 (42.8)	940 (47.9)	947 (47.5)	766 (43.7)	782 (42.3)
≥ 9	746 (43.9)	669 (42.1)	507 (25.8)	511 (25.6)	428 (24.4)	474 (25.6)
Maternal BMI (pre-gestational)^a^						
Normal	978 (57.3)	897 (56.4)	1480 (77.7)	1491 (76.2)	1181 (77.9)	1201 (76.3)
Overweight	486 (28.5)	446 (28.1)	325 (17.1)	366 (18.7)	266 (17.6)	297 (18.9)
Obese	242 (14.2)	247 (15.5)	101 (5.3)	99 (5.1)	69 (4.6)	77 (4.9)
Financing of delivery						
Public	1383 (81.0)	1311 (81.6)	1708 (86.9)	1734 (86.9)	1611 (91.9)	1678 (90.6)
Private	324 (19.0)	295 (18.4)	257 (13.1)	262 (13.1)	143 (8.1)	175 (9.4)
Birth weight (grams)						
< 2500	123 (7.2)	159 (9.9)	148 (7.5)	209 (10.5)	103 (5.9)	156 (8.4)
≥ 2500	1585 (92.8)	1450 (90.1)	1817 (92.5)	1787 (89.5)	1651 (94.1)	1696 (91.6)
Type of delivery						
Normal	905 (53.0)	905 (56.3)	1353 (68.9)	1376 (68.9)	1258 (71.7)	1348 (72.8)
C-section	803 (47.0)	704 (43.7)	612 (31.2)	620 (31.1)	496 (28.3)	505 (27.3)
Current continuous variables	Mean (sd)	Mean (sd)	Mean (sd)	Mean (sd)	Mean (sd)	Mean (sd)
Fat mass index (kg/m^2^)	3.3 (2.3)	4.2 (2.5)	4.2 (3.0)	8.4 (3.6)^a^	6.7 (3.2)^a^	10.6 (4.3)^a^
BMI Z-score	0.71 (1.5)	0.66 (1.4)	0.29 (1.2)^a^	0.45 (1.2)^a^	0.03 (0.9)^a^	−0.02 (1.1)^a^
Height (cm)	121.6 (5.6)	120.3 (5.6)	173.8 (6.9)	161.0 (6.4)	174.4 (6.9)	161.4 (6.2)
All	1708 (51.5)	1609 (48.5)	1965 (49.6)	1996 (50.4)	1754 (48.6)	1853 (51.4)

*Abbreviations: BMI* Body Mass Index

^a^For the 2004 cohort, we used information about maternal BMI three months after birth

Maximum percentage of unknown observations: *n* = 556 (14.3%) for maternal BMI (pre-gestational) in the 1982 cohort study; *n* = 99 (2.5%) for maternal BMI (pre-gestational) in the 1993 cohort study

Table 2 presents the results of our analyses for the 2004 birth cohort, with children at an average age of 6.8 years (min. 5.8 – max. 7.6 years). Fat mass index was strongly and positively associated with C-section in the crude analysis. In model 1, when we included socioeconomic characteristics (SEP, maternal education, skin colour and whether delivery was paid for private or through the public health system), the effect was reduced by 23–50% (β = 0.29; 95%CI 0.11; 0.47). When we adjusted for maternal BMI, parity, age and smoking during pregnancy, the effects were further reduced by 50–75% and the confidence interval included the null value (β = 0.10; 95%CI −0.08; 0.28). Adjustment for birth weight and length did not lead to further change in effect estimates.

**Table 2:** The 2004 cohort: crude and adjusted effects of cesarean section on fat mass index, BMI Z-score and height at age 6, overall and by sex. Adjusted effects obtained through linear regression. 2004 Pelotas Birth Cohort, Brazil, 2010–11

Type of delivery	All (*N* = 3317)	Males (*N* = 1708)	Females (*N* = 1609)
	Coeff. (CI 95%)	Coeff. (CI 95%)	Coeff. (CI 95%)
Fat mass index at age 6			
Crude effect	*p < 0.001*	*p < 0.001*	*p < 0.001*
C-section	0.51 (0.34;0.68)	0.60 (0.38;0.82)	0.48 (0.23;0.73)
Adjusted (model 1)^a^	*p = 0.002*	*p = 0.018*	*p = 0.008*
C-section	0.29 (0.11;0.47)	0.28 (0.05;0.52)	0.37 (0.09;0.64)
Adjusted (model 2)^b^	*p = 0.277*	*p = 0.532*	*p = 0.167*
C-section	0.10 (−0.08;0.28)	0.07 (−0.16;0.31)	0.18 (−0.08;0.44)
Adjusted (model 3)^c^	*p = 0.326*	*p = 0.574*	*p = 0.127*
C-section	0.09 (−0.09;0.27)	0.07 (−0.17;0.30)	0.20 (−0.06;0.46)
BMI Z-score at age 6			
Crude effect	*p < 0.001*	*p < 0.001*	*p < 0.001*
C-section	0.32 (0.22;0.42)	0.39 (0.25;0.53)	0.24 (0.11;0.38)
Adjusted (model 1)^a^	*p < 0.001*	*p = 0.007*	*p = 0.011*
C-section	0.20 (0.10;0.31)	0.21 (0.06;0.37)	0.19 (0.04;0.33)
Adjusted (model 2)^b^	*p = 0.083*	*p = 0.331*	*p = 0.166*
C-section	0.09 (−0.01;0.20)	0.08 (−0.08;0.23)	0.10 (−0.04;0.24)
Adjusted (model 3)^c^	*p = 0.138*	*p = 0.567*	*p = 0.139*
C-section	0.08 (−0.03;0.18)	0.04 (−0.11;0.20)	0.11 (−0.03;0.24)
Height (cm) at age 6			
Crude effect	*p < 0.001*	*p = 0.001*	*p = 0.002*
C-section	0.92 (0.53;1.30)	0.88 (0.35;1.40)	0.88 (0.32;1.43)
Adjusted (model 1)^a^	*p = 0.795*	*p = 0.839*	*p = 0.751*
C-section	0.05 (−0.35;0.46)	−0.06 (−0.62;0.50)	0.10 (−0.50;0.69)
Adjusted (model 2)^b^	*p = 0.161*	*p = 0.256*	*p = 0.253*
C-section	−0.29 (−0.71;0.12)	−0.33 (−0.90;0.24)	−0.35 (−0.94;0.25)
Adjusted (model 3)^c^	*p = 0.637*	*p = 0.391*	*p = 0.960*
C-section	−0.10 (−0.49;0.30)	−0.24 (−0.79;0.31)	−0.02 (−0.60;0.57)

^a^Model 1: adjusted for economic position at birth, maternal schooling, mother’s skin colour and financing of delivery

^b^Model 2: model 1 + mother’s BMI three months after the birth (linear and quadratic), parity, mother’s age at birth and smoking during pregnancy

^c^Model 3: model 2 + birth weight and birth length

We observed a very similar pattern of results for BMI-for-age z-score, with a strong crude association (β = 0.32; 95%CI 0.22; 0.42), and no association after adjustment for confounding variables (β = 0.08; 95%CI −0.03; 0.18). In the last section of the table, we observed the expected results for height – a strong crude association due to socioeconomic confounding (β = 0.92; 95%CI 0.53; 1.30) and no association in model 1, which introduces control for such variables (β = −0.10; 95%CI −0.49; 0.30) (Table 2).

In Table 3, we present the same analyses for the 1993 birth cohort, in which adolescents were, on average, aged 18.4 years (min. 17.8 – max. 19.2 years). In males, results for fat mass index were similar to those we found for the 6-year-old children. For females, no association was observed even in the crude analysis (β = 0.26; 95%CI −0.08; 0.60). Similarly, there was no association with BMI-for-age z-score after controlling for confounders for both males and females. The analysis for height showed no association, as expected, although the effects estimated for males and females were in opposite directions.

**Table 3:** The 1993 cohort: crude and adjusted effects of cesarean section on fat mass index, BMI Z-score and height at age 18, overall and by sex. Adjusted effects obtained through linear regression. 1993 Pelotas Birth Cohort, Brazil, 2011-12

Type of delivery	All (*N* = 3961)	Males (*N* = 1965)	Females (*N* = 1996)
	Coeff. (CI 95%)	Coeff. (CI 95%)	Coeff. (CI 95%)
Fat mass index at age 18			
Crude effect	*p = 0.001*	*p < 0.001*	*p = 0.137*
C-section	0.45 (0.18; 0.72)	0.66 (0.36; 0.96)	0.26 (−0.08; 0.60)
Adjusted (model 1)^a^	*p = 0.018*	*p = 0.009*	*p = 0.126*
C-section	0.34 (0.06; 0.62)	0.41 (0.10; 0.72)	0.28 (−0.08; 0.64)
Adjusted (model 2)^b^	*p = 0.364*	*p = 0.106*	*p = 0.806*
C-section	0.13 (−0.15; 0.41)	0.25 (−0.05; 0.56)	0.04 (−0.30; 0.39)
Adjusted (model 3)^c^	*p = 0.583*	*p = 0.123*	*p = 0.990*
C-section	0.08 (−0.20; 0.36)	0.24 (−0.07; 0.56)	−0.01 (−0.35; 0.34)
BMI Z-score at age 18			
Crude effect	*p < 0.001*	*p < 0.001*	*p = 0.209*
C-section	0.17 (0.09; 0.25)	0.27 (0.15; 0.38)	0.07 (−0.04; 0.19)
Adjusted (model 1)^a^	*p < 0.001*	*p = 0.001*	*p = 0.104*
C-section	0.15 (0.07; 0.24)	0.20 (0.08; 0.31)	0.10 (−0.02; 0.22)
Adjusted (model 2)^b^	*p = 0.082*	*p = 0.049*	*p = 0.599*
C-section	0.07 (−0.01; 0.16)	0.11 (0.00; 0.23)	0.03 (−0.09; 0.15)
Adjusted (model 3)^c^	*p = 0.163*	*p = 0.061*	*p = 0.840*
C-section	0.06 (−0.02; 0.14)	0.11 (−0.01; 0.23)	0.01 (−0.11; 0.13)
Height (cm) at age 18			
Crude effect	*p = 0.025*	*p = 0.293*	*p = 0.001*
C-section	0.70 (0.09; 1.32)	0.37 (−0.31;1.02)	1.04 (0.44;1.64)
Adjusted (model 1)^a^	*p = 0.684*	*p = 0.267*	*p = 0.042*
C-section	0.13 (−0.50;0.76)	−0.38 (−1.06;0.29)	0.64 (0.02; 1.27)
Adjusted (model 2)^b^	*p = 0.613*	*p = 0.045*	*p = 0.196*
C-section	−0.17 (−0.82; 0.48)	−0.71 (−1.40; −0.02)	0.42 (−0.22; 1.05)
Adjusted (model 3)^c^	*p = 0.932*	*p = 0.080*	*p = 0.130*
C-section	−0.02 (−0.65; 0.59)	−0.59 (−1.25; 0.07)	0.47 (−0.14; 1.09)

^a^Model 1: adjusted for economic position at birth, maternal schooling, mother’s skin colour and financing of delivery

^b^Model 2: model 1 + mother’s pre-gestational BMI (linear and quadratic), parity, mother’s age at birth and smoking during pregnancy

^c^Model 3: model 2 + birth weight and birth length

Table 4 shows the results for the 1982 cohort, in which participants were, on average, aged 30.2 years (min. 29.4 – max. 31.1 years). Results for males followed the same pattern described before for the three indicators assessed in our study (BMI z-score, fat mass index and height). Nevertheless, C-section remained associated with BMI z-score and fat mass index in women, even after adjustment for confounders (β for fat mass index = 0.82; 95% CI 0.32;1.32, and β for BMI z-score = 0.15; 95% CI 0.03;0.28). Differently from all the other sets of analysis, we did not observe reductions in the magnitude of the associations in the adjusted models for women. In this case, the *p*-value for the association between C-section and height was borderline (p = 0.053).

**Table 4:** The 1982 cohort: crude and adjusted effects of cesarean section on fat mass index, BMI Z-score and height at age 30, overall and by sex. Adjusted effects obtained through linear regression. 1982 Pelotas Birth Cohort, Brazil, 2012–13

Type of delivery	All (*N* = 3607)	Males (*N* = 1754)	Females (*N* = 1853)
	Coeff. (CI 95%)	Coeff. (CI 95%)	Coeff. (CI 95%)
Fat mass index at age 30			
Crude effect	*p < 0.001*	*p < 0.001*	*p < 0.001*
C-section	0.68 (0.35;1.00)	0.58 (0.23;0.93)	0.90 (0.44;1.36)
Adjusted (model 1)^a^	*p < 0.001*	*p = 0.021*	*p < 0.001*
C-section	0,66 (0.33;0.99)	0.41 (0.06;0.77)	1.10 (0.63;1.57)
Adjusted (model 2)^b^	*p = 0.077*	*p = 0.621*	*p = 0.001*
C-section	0.32 (−0.03;0.68)	0.09 (−0.28;0.47)	0.82 (0.32;1.32)
Adjusted (model 3)^c^	*p = 0.075*	*p = 0.619*	*p = 0.001*
C-section	0.32 (−0.03;0.68)	0.10 (−0.28;0.47)	0.82 (0.32;1.32)
BMI Z-score at age 30			
Crude effect	*p < 0.001*	*p = 0.012*	*p = 0.015*
C-section	0.13 (0.06;0.21)	0.12 (0.03;0.21)	0.14 (0.03;0.26)
Adjusted (model 1)^a^	*p < 0.001*	*p = 0.065*	*p < 0.001*
C-section	0.15 (0.08;0.23)	0.09 (−0.01;0.18)	0.22 (0.10;0.34)
Adjusted (model 2)^b^	*p = 0.021*	*p = 0.426*	*p = 0.018*
C-section	0.09 (0.01;0.17)	0.04 (−0.06;0.14)	0.15 (0.03;0.28)
Adjusted (model 3)^c^	*p = 0.019*	*p = 0.415*	*p = 0.018*
C-section	0.09 (0.01;0.17)	0.04 (−0.06;0.14)	0.15 (0.03;0.28)
Height (cm) at age 30			
Crude effect	*p = 0.399*	*p = 0.527*	*p = 0.947*
C-section	0.29 (−0.39;0.97)	0.23 (−0.49;0.96)	0.02 (−0.62;0.67)
Adjusted (model 1)^a^	*p = 0.383*	*p = 0.193*	*p = 0.054*
C-section	−0.31 (−0.99;0.38)	−0.47 (−1.18;0.24)	−0.63 (−1.27;0.01)
Adjusted (model 2)^b^	*p = 0.602*	*p = 0.150*	*p = 0.068*
C-section	−0.21 (−0.96;0.55)	−0.57 (−1.36;0.21)	−0.65 (−1.35;0.05)
Adjusted (model 3)^c^	*p = 0.560*	*p = 0.145*	*p = 0.053*
C-section	−0.22 (−0.95;0.51)	−0.56 (1.32;0.19)	−0.67 (−1.34;0.01)

^a^Model 1: adjusted for economic position at birth, maternal schooling, mother’s skin colour and financing of delivery

^b^Model 2: model 1 + mother’s pre-gestational BMI (linear and quadratic), parity, mother’s age at birth and smoking during pregnancy

^c^Model 3: model 2 + birth weight (birth length is not available for this cohort)

## Discussion

In this analysis of three Brazilian birth cohorts, our results are very clear in showing no consistent association of C-section with either BMI z-score or the directly measured fat mass index. For the 1993 and 2004 cohorts, and the males of the 1982 cohort, the strong association seen in the crude model became null after adjusting for confounders, especially maternal pre-gestational BMI and socioeconomic characteristics. On the other hand, for females in the 1982 cohort, C-section remained associated with BMI z-score and fat mass index, even after adjustment for confounders. This isolated association, along with the borderline association between C-section and height for the same group seems most likely to be a result of residual confounding, since there is no apparent reason for a specific effect within this subgroup.

The relationship between C-section and obesity based on BMI had already been studied in these same Brazilian cohorts at ages of 4, 11, 15 and 23 years [17]. In these previous analyses, no consistent association between C-section and obesity was found after adjustment for the same confounders included in our model. Also consistent with our analyses, there was an increased effect of C-section on obesity among 23-year-old women from the 1982 cohort after adjustment for confounders. But previously obesity was treated as a dichotomous outcome (compared to the continuous outcomes used in our analysis), and there was no available information about direct measures of body fat. In our analysis, in 30-year-old women from the 1982 cohort, results remained significant, probably because of increased statistical power due to the use of continuous outcomes.

Despite some studies corroborating our findings by showing a null association between C-section and BMI [12, 17], the evidence in the literature is, so far, conflicting, given that several other studies found positive associations. A study conducted in Denmark with a sample of adults found that C-section was associated with a higher prevalence of obesity in men but not in women [8]. Goldani et al. [6], studying Brazilian adults, found that those born by C-section had 58% higher risk of obesity. Another Brazilian study found a consistent increase in BMI among children born by C-section in two different regions of the country [26]. In addition, Huh et al.[7] found that children born by C-section showed higher odds of obesity at age 3 years.

Two recent meta-analyses [11, 27] investigated the association between C-section and overweight/obesity. Lih et al. [27] concluded that C-section increases the risk of overweight and obesity by 33%. However, it is important to highlight that the positive results seen in this meta-analysis were observed in smaller sample size studies and, in most cases, in those with medium quality. Kuhle et al. [11] observed that C-section increased the risk of overweight and obesity by 34% in children, but the risk was higher in those studies with no adjustment for maternal weight in the pre-pregnancy period.

Several factors can explain the conflicting results among studies, such as differences in body mass distribution and prevalence of obesity in different settings as well as differences in C-section prevalence and C-section patterning among population strata of different countries. Furthermore, some studies that indicated a positive association did not control for SEP or maternal pre-gestational BMI [6, 8], which are important confounders in the association between C-section and obesity, as we have confirmed in our analyses.

The link between C-section, obesity and gut microbiota is biologically plausible. However, it is currently not entirely clear whether gut microbiota leads to obesity or vice-versa. In addition, there is evidence that gut microbiota at birth changes across the life course as a result of several factors, including the use of antibiotics and diet [28, 29]. In any case, the control of potential confounders is of paramount importance in studies investigating the association of C-section and obesity.

In countries with low C-section rates, where medical indication is the main determinant of the intervention, large babies and obese mothers are certainly one important determinant of C-section deliveries. Therefore, adjusting for maternal BMI and baby size is essential. In countries where C-section rates are high (the case of Brazil, where C-section rates has increased in recent years), rich women are much more likely to have a C-section. We have clear evidence that rich children are fatter than poor children and that rich men are fatter than poor men [30, 31]. Among women, the association is reversed in some sites but not in others [3, 32, 33]. Again, controlling for socioeconomic position and maternal BMI is essential to achieve an unbiased estimate of the association between C-section and offspring excess weight.

With our three birth cohort studies, we are in a strong position to study this association since we have detailed body composition data in a large number of participants at different ages (6, 18 and 30 years). Instead of relying solely on BMI to assess obesity, an indicator that has some limitations, we have direct estimates of body fat mass. Moreover, we have collected extensive information on characteristics that are important potential confounders, including socioeconomic and anthropometric data from mothers and offspring. The main limitations of our study are related to how wealth was measured in the 1982 cohort. Instead of using assets, income was recorded in five categories, and only afterwards more detailed information was gathered.

## Conclusion

In conclusion, there was no consistent association of C-section with BMI, fat mass index, measured by DXA, or height in individuals aged 6, 18 and 30 years. Given the quality of our body composition data, the inclusion of several ages and the high (and increasing) C-section rates in Brazil, we add strong evidence against the association between C-section and increased adiposity.

## Abbreviations

BMI: Body mass index
CI: Confidence interval
C-Section: Caesarian section
DXA: Dual-energy X-ray absorptiometry
IEN: Brazilian National Economic Index
SEP: Socioeconomic position
USA: United States of America.
